# Transdermal administration of melatonin coupled to cryopass laser treatment as noninvasive therapy for prostate cancer

**DOI:** 10.1080/10717544.2017.1338793

**Published:** 2017-06-23

**Authors:** Laura Terraneo, Paola Bianciardi, Eleonora Virgili, Elena Finati, Michele Samaja, Rita Paroni

**Affiliations:** Department of Health Science, University of Milan, Milano, Italy

**Keywords:** Melatonin, drug delivery, experimental prostate cancer, cryopass-laser therapy, anticancer activity, transdermal administration

## Abstract

Melatonin, a pineal gland hormone, exerts oncostatic activity in several types of human cancer, including prostate, the most common neoplasia and the third most frequent cause of male cancer death in the developed world. The growth of androgen-sensitive LNCaP prostate cancer cells in mice is inhibited by 3 mg/kg/week melatonin (0.09 mg/mouse/week) delivered by i.p. injections, which is equivalent to a dose of 210 mg/week in humans. The aim of this study is to test an alternative noninvasive delivery route based on transdermal administration of melatonin onto the tumor area followed by cryopass-laser treatment. Two groups of immunodepressed mice were studied, one (*n* = 10) subjected to 18 cryopass-laser therapy sessions and one (*n* = 10) subjected to the same treatment without melatonin. These groups were compared with mice treated with i.p.-administered melatonin or vehicle with the same time schedule. We found that cryopass-laser treatment is as efficient as i.p. injections in reducing the growth of LNCaP tumor cells, affecting plasma melatonin and redox balance. Furthermore, both delivery routes share the same effects on the involved biochemical pathway driven by hypoxia-inducible factor 1α. However, cryopass-laser, as used in the present experimental setup, is less efficient than i.p delivery route in increasing the melatonin content and Nrf2 expression in the tumor mass. We conclude that cryopass-laser treatment may have impact for melatonin-based therapy of prostate cancer, by delivering drugs transdermally without causing pain and targeting directly on the site of interest, thereby potentially making long-term treatments more sustainable.

## Introduction

Prostate cancer affects one in five of all newly diagnosed cases of male cancers and is the third cause of cancer-related death among men (Siegel et al., 2017). The vast majority of prostate cancers are diagnosed at an early stage, but approximately 15% of men with newly diagnosed prostate cancer display high-risk disease with metastatic progression and poor outcome (Miller et al., 2016, Wang et al., 2016a). Most patients show favorable initial response to androgen deprivation therapy or castration, but in the long term almost all patients develop progression from androgen-dependent to more aggressive androgen-independent stage with development of metastases and decreased quality of life. Although chemotherapy improves survival, its side effects on healthy cells and the linked cytotoxicity limit its use especially in older patients (Poorthuis et al., 2017). Clinical studies have demonstrated that the supplementation of melatonin may enhance the efficacy and reduce the side effects of chemotherapy, prolonging survival and improving the quality of life (Lissoni et al., 2006; Bizzarri et al., 2013; Ma et al., 2016; Najafi et al., 2017).

A natural molecule secreted by the pineal gland especially nighttime, melatonin (N-acetyl-5-methoxy tryptamine) serves as a bio-clock regulator of an array of physiological functions (Kelleher et al., 2014) and displays almost null toxicity (Flo et al., 2016). Melatonin has paracrine, autocrine as well as antioxidant effects, and exerts diverse receptor-dependent and receptor-independent actions, with overall homeostatic functions and pleiotropic effects relevant to cell protection and survival (Srinivasan et al., 2008; Luchetti et al., 2010). Melatonin is known to display oncostatic activity in a variety of tumors including breast (Mao et al., 2016), ovarian (Zhao et al., 2016), colon (Gao et al., 2016), endometrial (Ciortea et al., 2011), gastrointestinal (Wang et al., 2016b), and prostate (Paroni et al., 2014). The link between plasma melatonin and prostate cancer risk is well recognized. The decline in melatonin production with age was suggested as a major contributor of the development of cancer in elder people (Srinivasan et al., 2011; Hill et al., 2013). Furthermore, shift workers have an increased risk for prostate cancer (Dumont et al., 2012), and exposure to artificial light at night is associated with prostate cancer because it disturbs endogenous circadian rhythms leading to the suppression of nocturnal melatonin production (Kim et al., 2016). Melatonin likely affects tumor biology via multiple mechanisms that include the modulation of the redox balance, immune system, angiogenesis, endocrine system, androgen receptors signaling, as well as the direct action via specific membrane receptors (Tam and Shiu, 2011; Gonzalez et al., 2017). *In vitro* and *in vivo* models document that melatonin displays a relevant antiproliferative activity in cancer (Bizzarri et al., 2013). In immunodepressed mice treated with melatonin (18 i.p. injections of 1 mg/kg melatonin for 42 days, 3 injections/week), the growth of LNCaP prostate cancer was reduced 4-fold compared to saline-treated control (Paroni et al., 2014). The marked antitumor activity of melatonin even without association with any other drug calls the opportunity to investigate novel roads for alternative painless and noninvasive ways of administering this substance to improve the sustainability of such therapy for long-term treatments. Here, we test the antiproliferative activity of melatonin when delivered by cryopass-laser treatment, a noninvasive transdermal administration technology suitable for local delivery of drugs into specific areas potentially avoiding distribution in non-target tissues and unwanted systemic effects. We test this approach in the same murine model that proved useful to assess the antitumor effect of melatonin given via i.p. injections.

## Methods

### Mice and experimental design

Seven-week old Foxn1^nu/nu^ mice (Harlan, *n* = 20), weighing 25–30 g at the entry into the study, were cared in accordance to the Guide for the Care and Use of Laboratory Animals published by the National Institutes of Health (NIH Publication No. 85-23, revised 1996). The Ethical Committee of the University of Milan approved the experimental protocol (All.5verb.16.03.2010). Water and bedding were heat-sterilized, whereas food was sterilized by ^60^Co γ-irradiation. Mice had free access to water and food until 24 h before sacrifice. A12/12 h light/dark cycle was maintained.

**Figure 1.**
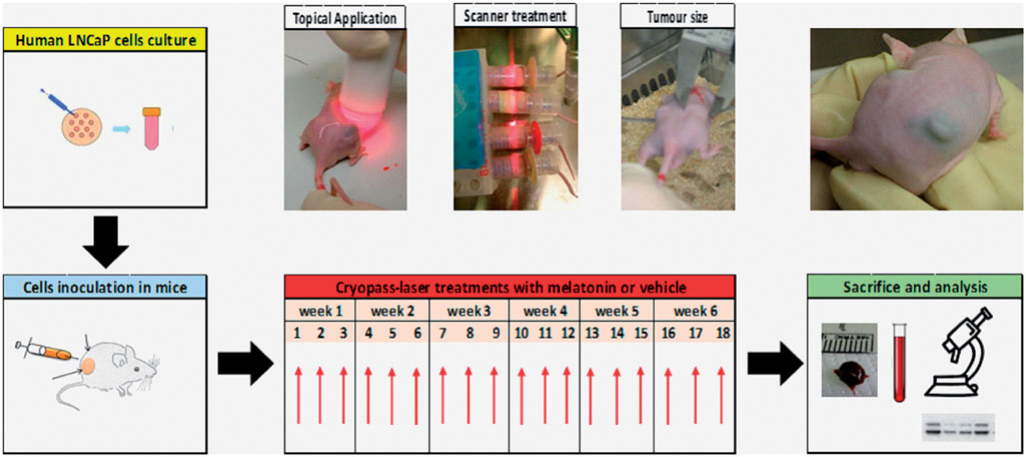
Experimental flowchart. Human prostate cancer cells (LNCaP) were cultured and resuspended in ice-cold Matrigel (1:1) at a final concentration of 3x106/0.1 ml. Mice were inoculated in each flank with LNCaP and subjected to cryopass-laser treatment (with 4 mg/kg melatonin or vehicle) three times/week, for 6 weeks, for a total of 18 treatments. At the end of the experimental time, tumors and blood were collected for the biochemical analysis.

LNCaP cells (ATCC) maintained in RPMI-1640 medium (Euroclone), were resuspended in Matrigel (1:1) and inoculated in each flank of mice (3 × 10^6^ cells/0.1 ml). Mice were then randomized and subjected to cryo-pass laser treatment with either vehicle (vehicle-laser, *n* = 10) or melatonin (melatonin-laser, *n* = 10). Body weight and tumor volume were measured three times a week for 42 days. At the end of the observation period, mice were anesthetized, thoracotomized to withdraw a blood sample, and tumors were quickly excised from surrounding skin and frozen as described (Paroni et al., 2014). Figure 1 shows the flowchart of the experimental design.

### Cryopass-laser treatment

The equipment for cryopass-laser treatment (LASERICE Med C.I.R.C.E. S.r.L., Magnago, Milano) is constituted by cryo applicators containing frozen emulsions with 1.5% (w/v) hydroxymethyl cellulose with or without 0.048 mg melatonin/ml, and by a scanner connected to a photodiode laser beam generator with λ = 635 nm, maximum power <5 mW, collimation lens <20 mV. The preparation of the cryo applicators took place before the beginning of the experiment. The suspension was prepared by emulsifying for 7 min in ice and dark with a disperser tool (Ultra-Turrax T25, IKA Labortechnik, Staufen, Germany) at the maximum speed. Then, 15 ml of this suspension was transferred into cryo applicators (0.72 mg melatonin each) and they were immediately frozen at −20 °C until use. A single cryo applicator was used to treat six mice, therefore the amount of administered melatonin was ∼0.120 mg melatonin/mouse/treatment, or ∼4 mg/kg. For the administration, the cryo applicator was connected to a laser beam source and subsequently melted by rubbing on the mice’s flanks in the correspondence of the area where LNCaP cells were inoculated. The duration of the treatment was 2.4 min/mice. As the application of frozen sticks may cause hypothermia, mice were kept on a heating plate at 37 °C. After this phase, mice were placed in specially devised constrictors specially assembled to expose the inoculated area on the animal flanks. Then animals were subjected to a high-power laser scanner connected to a laser beam generator that oscillated over them for 15 min. Cryo-laser application over the skin area of the inoculum, was performed three times/week for 42 days to a total of 18 treatments.

### Biochemical measurements

After sacrifice (day 42), we measured blood hemoglobin (Hb) concentration (Terraneo et al., 2014), oxidative capacity in plasma (d-ROMS test, Diacron International srl, Grosseto, Italy), the plasma antioxidant capacity (Total Antioxidant Capacity Assay kit Catalog #K274-100; BioVision, Inc., Mountain View, CA, USA), as well as plasma and tumor contents of melatonin (Melatonin ELISA REF RE54021; IBL, Hamburg, Germany) as described (Paroni et al., 2014).

The expression of selected proteins was measured in tumor biopsies by Western blot (Terraneo et al., 2014). The primary antibodies and dilutions were: anti-HIF-1α (inducible factor-1alpha, Santa Cruz Biotechnology, 1:300), anti-Nrf2 (nuclear factor (erythroid-derived 2)-like 2, Santa Cruz Biotechnology, 1:1000), anti-β-actin (Sigma Aldrich, St Louis, MI 1:5000), The secondary antibodies were horseradish peroxidase-conjugated anti-mouse IgG (Jackson Immuno Research, West Grove, PA, 1:10,000) or anti-rabbit IgG (Jackson Immuno Research, West Grove, PA, 1:10,000). Chemiluminescence was detected by incubating the membrane with LiteAblot Chemiluminescent substrate (Lite Ablot, EuroClone, EMPO10004). After the images were acquired using the Alliance LD6 image capture system (UVITEC Cambridge Ltd, UK). Densitometric analysis was performed using UVI-1 D software (UVITEC Cambridge Ltd, UK). The HIF-1α level was also assessed by immunofluorescence assay, by summing the green pixels intensities in 4–5 microphotographs taken from each image, exclusively on tumor area, excluding the areas related to inflammatory infiltrate (Terraneo et al., 2014).

### Statistical analysis

Data are reported as mean ± SEM. To facilitate the comparison between the effects of two routes of administration, data related to melatonin or vehicle delivered via i.p. (melatonin-ip and vehicle-ip groups) obtained in a previously published study (Paroni et al., 2014) are also reported. The previously published and the present studies were performed using the same analysis procedures and timings, and differ only for the treatments, i.p. or cryopass laser. Thus, we performed two-way analysis of variance (ANOVA) assuming two factors: the delivery route (i.p. or laser) and the treatment (melatonin or vehicle). Statistics was performed using GraphPad Prism 6 software (GraphPad Software, Inc.), with the significance level set at *p* = .05.

## Results

### Safety of cryopass-laser treatment

**Figure 2.**
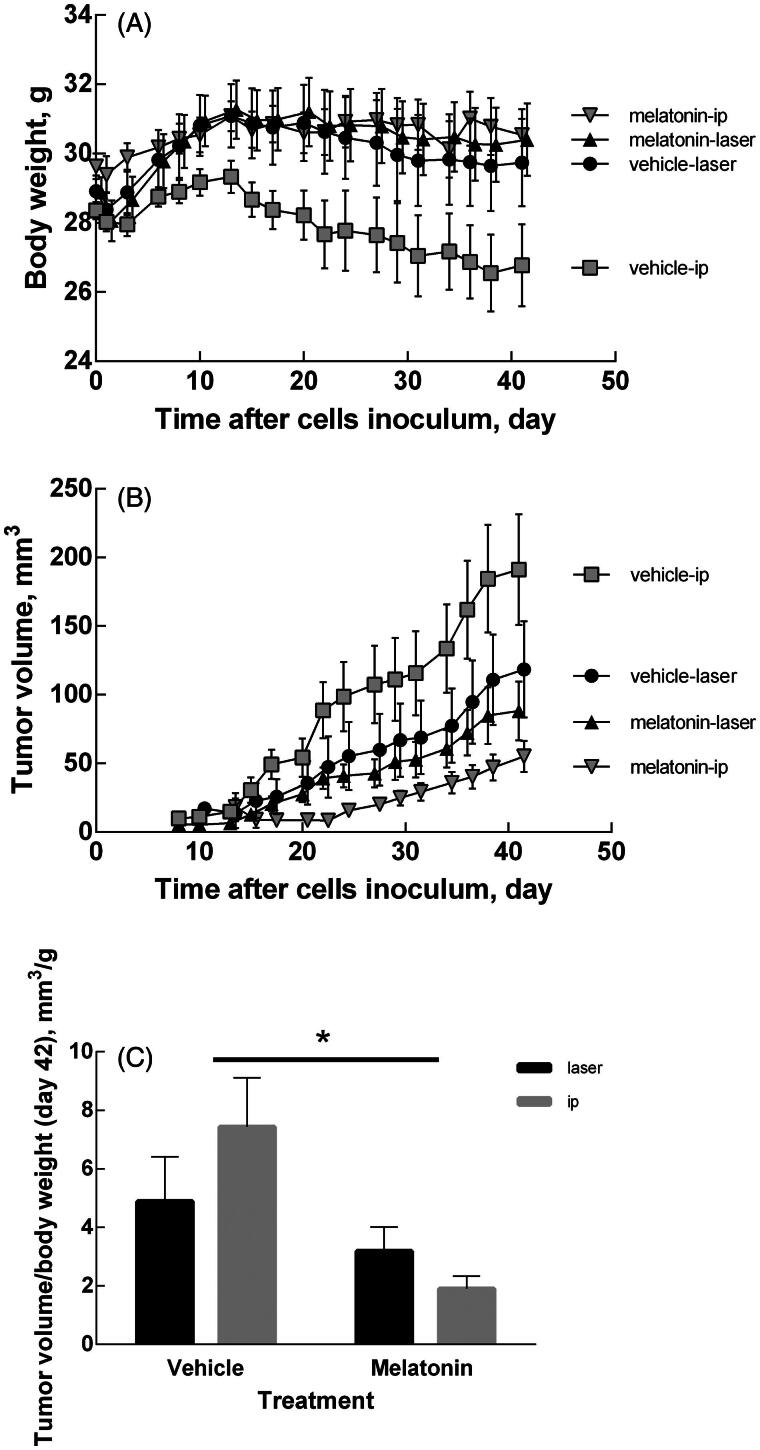
Body weight and tumor volume changes. (A) Time course of body weight of mice treated with melatonin or vehicle. (B) Time course of tumor volume in mice treated with melatonin or vehicle. Tumor volume was calculated as length x width x height x 0.5236 by a caliper. (C) Tumor volume at day 42. Data are expressed as the ratio (tumor volume)/(body weight) to compensate different rates of body growth in the experimental groups. Data are expressed as mean ± SEM, *p < .05 for treatment factor (Two-way ANOVA).

All the mice enrolled in this study survived without adverse effects. Despite their thin skin, none of the treated mice exhibited signs of skin burns. When melatonin was delivered by cryopass-laser treatment, the body weight increased similarly to previous experiments when melatonin was delivered via i.p. administration (Figure 2(A)). No differences in Hb concentration were detected among the groups (7.77 ± 0.66, 7.53 ± 0.23, 7.41 ± 0.34, 7.36 ± 0.37 mM respectively, for vehicle-laser, vehicle-ip, melatonin-laser, melatonin-ip).

### Melatonin inhibited LNCaP tumor growth independently of the delivery route

Figure 2(B) shows the time course of the tumor volume during the experimental time in all groups under study. Figure 2(C) reports the tumor volume measured on the last day of treatment. Two-way ANOVA shows that tumor growth was affected by the treatment (*p* = .0018) but not by the delivery route (*p* = ns). The interaction of these two factor was not significant (*p* = ns).

### Laser-melatonin treatment induces changes in plasma

**Figure 3.**
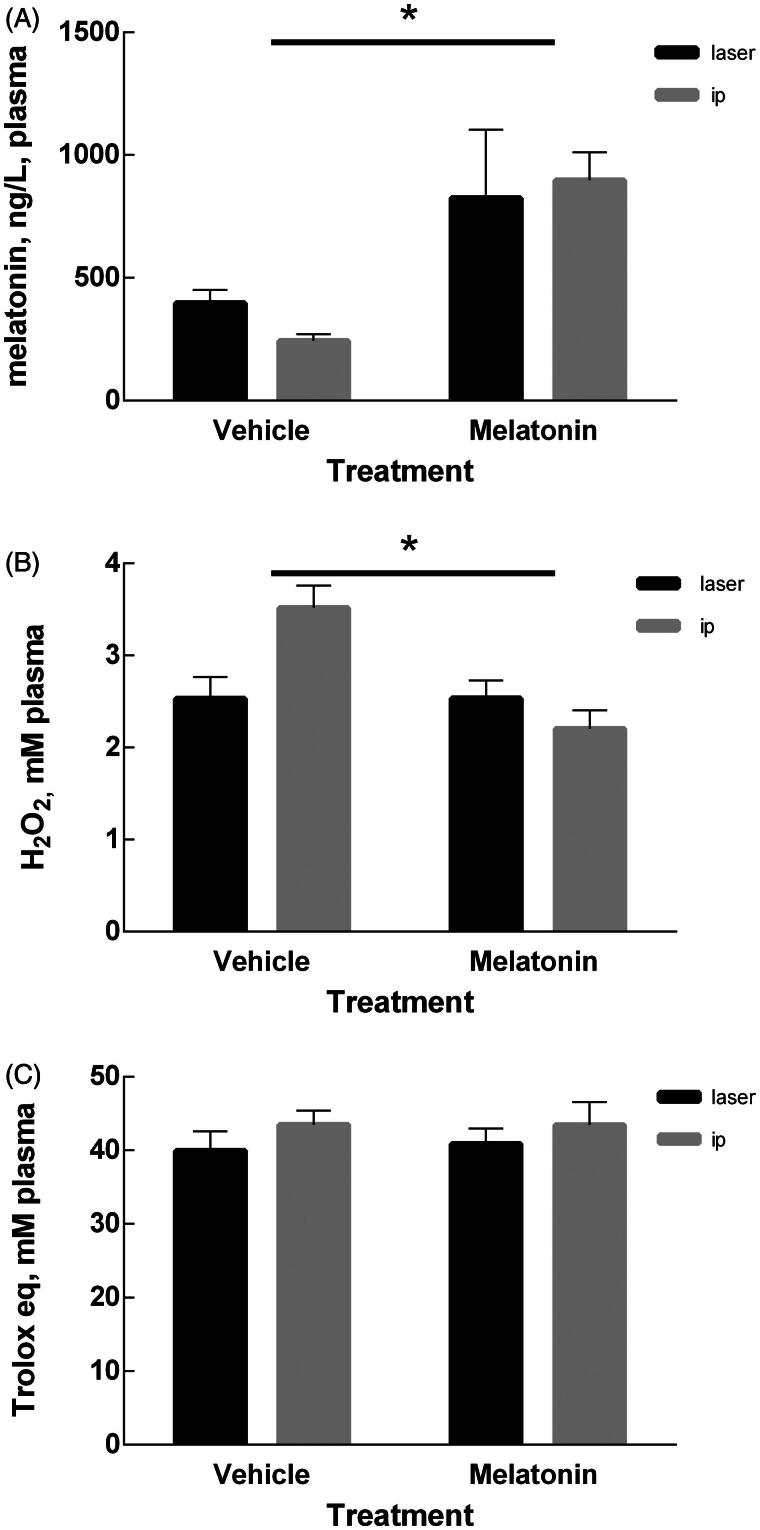
Plasma measurements. (A) Melatonin content in plasma at day 42 of mice treated with melatonin or vehicle. (B) Oxidant capacity in plasma determined measuring Reactive Oxygen Metabolites (ROMs) and expressed as H_2_O_2_ equivalents. (C) Plasma antioxidant capacity expressed as Trolox equivalents. Data are expressed as mean ± SEM, *p < .05 for treatment factor (two-way ANOVA).

Figure 3(A) reports the melatonin plasma levels in the four groups. Two-way ANOVA shows that the plasma level of melatonin was affected by the treatment (*p* = .0004) but not by the delivery route (*p* = ns). The interaction of these two factors was non-significant (p = ns). Likewise, the redox imbalance in plasma was affected by the treatment (*p* = .0072) independently of the delivery route (*p* = ns), but in this case the interaction of the two factors was significant (*p* = .0068) (Figure 3(B)). Finally, the plasma antioxidant capacity remained unchanged by either factor (Figure 2(C)).

### Melatonin administration by cryopass-laser and by i.p. share the same biochemical pathways

**Figure 4.**
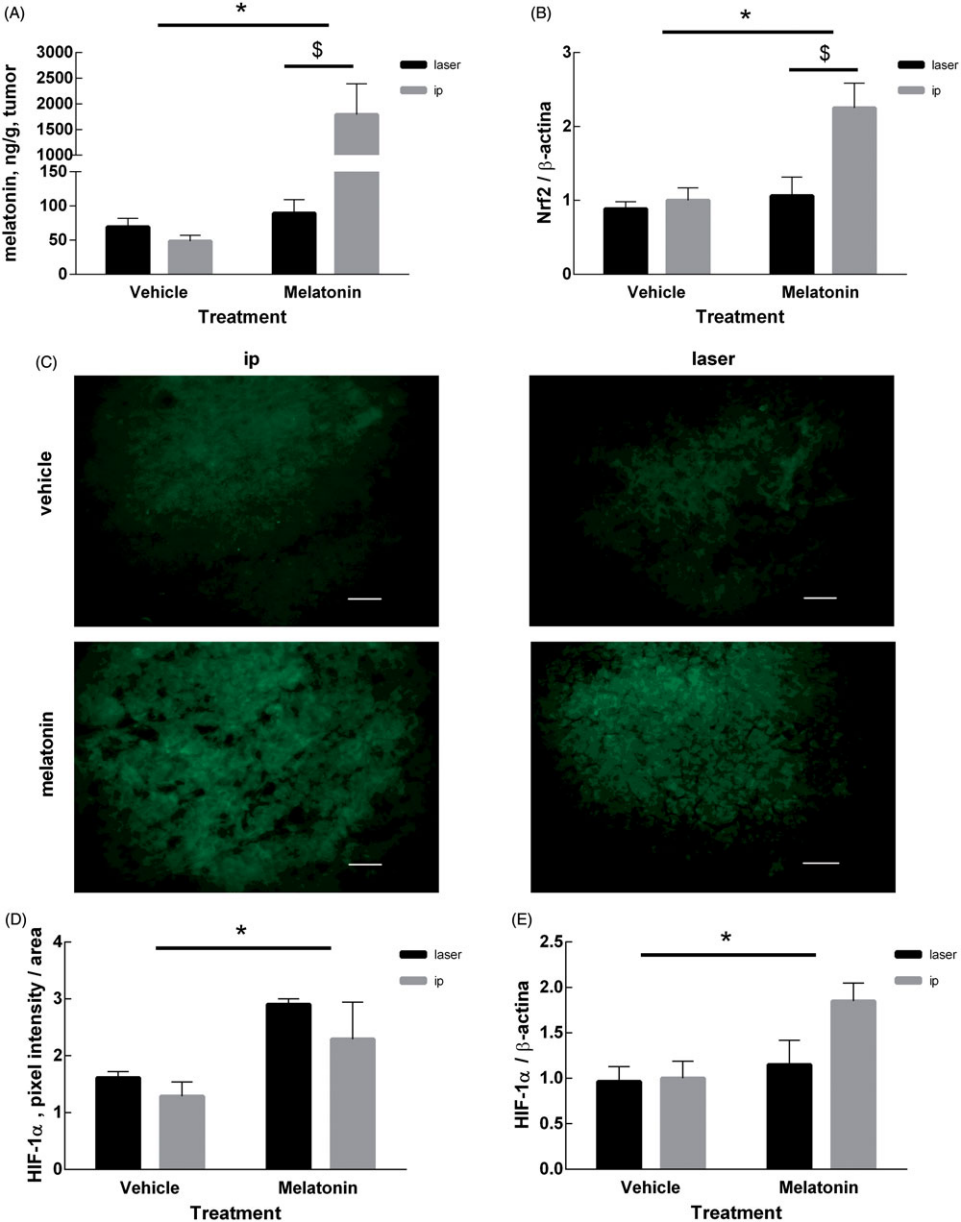
Measurements in tumor mass. (A) Melatonin content in tumor of mice treated with melatonin or vehicle, determined by competitive enzyme immunoassay as described in the Methods section. (B) Expression of Nrf2 protein measured by Western Blot. The intensity of Nrf2 bands were quantified and expressed as ratio with the intensity of β-actin bands. (C) Representative microphotographs of HIF-1α marked by immunofluorescence of all the experimental groups considered. The bars represent 50 μm. (D) Quantification of the HIF-1α signal measured as the sum of green pixels intensities exclusively in the tumor area, without considering the inflammatory infiltrate area. (E) Expression of the HIF-1α protein measured by Western Blot. The intensity of HIF-1α bands were quantified and expressed as ratio with the intensity of β-actin bands. Data are expressed as mean ± SEM, *p < .05 for treatment factor (Two-way ANOVA) and ^$^*p* < .05 for delivery route factor.

The melatonin content in tumor tissue was affected by both the treatment (*p* < .0001) and the route of administration (*p* < .001). The interaction of the two factors was extremely significant (*p* < .0001) in reducing tumor size (Figure 4(A)). To assess whether the antitumor effect of melatonin follows the same biochemical pathways independently of the delivery route, we measured two markers that were found to be altered by i.p. melatonin. Figure 4(B) shows the effects of treatment and delivery route on the expression of Nrf2, a protein activated in response to oxidative insult. Two-way ANOVA shows that Nrf2 is affected by both treatment (*p* = .0109) and delivery route (*p* = .0185), with significant interaction of the two factors (*p* = .0466). This indicates that the antitumor effect of melatonin is independent of the route of administration and it is mediated in part by the known antioxidant activity of melatonin.

Figure 4(C–E) show the effects of treatment and delivery route on the expression of HIF-1α, that was measured by immunofluorescence techniques (Figure 4(C) and (D)) and Western blotting (Figure 4(E)). Both techniques converged in indicating that HIF-1α expression was affected by the treatment (*p* = .0104 and *p* = .00308, respectively) but not by the delivery route (*p* = ns for either case). The interaction of the two factors was not significant (*p* = ns for either case).

## Discussion

The transdermal administration of melatonin by the described cryopass-laser treatment proved to be efficient to reduce the growth of LNCaP cells. The experimental setup designed for cryopass-laser and i.p. treatments was rigorously the same, with the only difference of the ways and amounts of melatonin administrations. Cryopass-laser treatment revealed to be a safe procedure without measurable side effect as outlined by similar rates of body weight increase and blood Hb content at the end of the observation. Both delivery routes significantly decreased the growth of LNCaP cells. In addition, both delivery routes affected by similar extents the plasma melatonin level and the redox imbalance, without altering the antioxidant capacity.

Early work already reported beneficial effects for transdermal delivery of melatonin (Lee et al., 1994). For example, transdermal melatonin delivery through patches can elevate the plasma melatonin level for an extended duration thereby improving sleep maintenance to a greater extent than melatonin per os (Aeschbach et al., 2009). Transdermal melatonin may be advantageous with respect to per os delivery especially in elderly patients because of reduced age-driven gastroenteric absorption (Flo et al., 2016). Furthermore, per os administration might imply poor bioavailability due to high liver metabolism and short plasma half-life of melatonin (Babu et al., 2008). However, it must be pointed out that substance penetration across the derma might also depend strikingly on the inter-subject variability of stratum corneum ultrastructure and composition, race and color of skin, temperature across skin, in addition to dose and surface of application (Oh et al., 2001; Aeschbach et al., 2009; Flo et al., 2016; Marwah et al., 2016). Although both the stratum corneum and epidermis of skin may block the penetration of drugs, Figure 3(A) shows that the cryopass-laser treatment enables marked increase of the plasma level of melatonin.

The cryopass-laser treatment is a procedure that promotes the delivery of a drug molecule across the dermal barrier in substitution of either chemical permeation enhancers, which could irritate the skin (Andréo et al., 2016), or electric current application by means of electrodes, which could be painful and cause skin burns. The cryopass-laser technology promotes biophysical permeation of drugs and, in contrast with the application of electrodes, is suitable for polar and non-polar molecules. The depth reachable by molecules in the target tissue can be adjusted by changing the power of the laser beam and the duration of the treatment. The developed technique is based on the effect of a laser bean and is less traumatic and painful, better targetable and more specific than chemical permeation and electrophoresis therapy. In the described application, an electromagnetic-wave generator (Bonizzoni, 2007) emits the energy needed for the process that does not damage the skin: despite the thinness of their skin, none of the treated animals exhibited burns. The reason why the molecule need to be frozen in the cryo-applicators resides in the laws of quantum physics. According to this theory, when a photon hits an electron in the outer orbital of the molecule, the applied energy excites the electron and makes it to jump to the higher energy level. The subsequent decay process to its initial level with re-emission of the photon is relatively slow when the molecule is in the frozen state. In other words, the lower the temperature, the slower the decay, which leads to energy conservation in the form of potential energy. This facilitates the release of energy in the form of kinetic energy only when the frozen molecule melts at the temperature of the ice-skin interface, thereby speeding up the passage of the drug across the skin to the target area. Once the drug molecules have penetrated the skin, a second laser scanning carried out on the region to be treated enables better subdermal distribution of the drug and facilitates targeting over the site of activity.

The cryopass-laser technology has been successfully used in animal model of spinal cord lesion (de Souza et al., 2013) and in several clinical uses.

Despite all the characteristics of LNCaP cells inoculation were rigorously the same for either routes of melatonin administration, the choice to compare two different delivery routes necessarily implies that the doses of melatonin can’t be the same as it could be desirable. Thus, the dose of melatonin used in the i.p. experiments (1 mg/kg) is not comparable with the dose of 4 mg/Kg, or 0.12 mg melatonin/mouse for each treatment, in the cryopass-laser treatments. Therefore, it is not surprising that the decrease of the tumor growth rates was not coincident for the two delivery routes, and we can’t exclude that adjusting the dose of melatonin or the number of treatments could narrow the remaining difference between the delivery routes. Nevertheless, in either case melatonin displays not only similar antitumor capacity, but also similar recruitment of the involved biochemical signaling pathways. Indeed, it appears that melatonin delivery via cryopass-laser treatment is as efficient as melatonin delivery via the i.p. route in reducing the growth of LNCaP tumors. Furthermore, either administration routes affect similarly the antioxidant response and HIF-1α overexpression. Similar recruitment of the basic mechanisms induced by melatonin may make the cryopass-laser technology a valid candidate for treatment of a life-threatening disease.

## Conclusions

Besides confirming the beneficial effects of melatonin on LNCaP tumor growth and providing the proof of concept for an application in human models, this study emphasizes the possibility to devise alternative ways to deliver melatonin in clinical contexts. In facts, inefficient or unspecific drug delivery to the site of action is a well-known limitation in several therapies. Here, we propose the transdermal administration of the natural molecule melatonin coupled to cryopass laser treatment but this approach could be suitable also with more potent anticancer drugs to be used as painless therapy reducing systemic effects to a minimum, thereby making it more sustainable for long-term treatments.
